# The Functions and Applications of RGD in Tumor Therapy and Tissue Engineering

**DOI:** 10.3390/ijms140713447

**Published:** 2013-06-27

**Authors:** Fen Wang, Yuanyuan Li, Yingqiang Shen, Anming Wang, Shuling Wang, Tian Xie

**Affiliations:** 1 College of Material Chemistry and Chemical Engineering, Hangzhou Normal University, Hangzhou 310036, China; E-Mails: wangfen@126.com (F.W.); liyuanyuan43@live.com (Y.L.); 2 Research Center for Biomedicine and Health, Hangzhou Normal University, Hangzhou 311121, China; E-Mails: shenyq@126.com (Y.S.); wsling222@163.com (S.W.); 3 College of Biological and Environmental Sciences, Hangzhou Normal University, Hangzhou 310036, China

**Keywords:** RGD, tumor therapy, tissue engineering

## Abstract

Arginine-Glycine-Aspartic (RGD), is the specific recognition site of integrins with theirs ligands, and regulates cell-cell and cell-extracellular matrix interactions. The RGD motif can be combined with integrins overexpressed on the tumor neovasculature and tumor cells with a certain affinity, becoming the new target for imaging agents, and drugs, and gene delivery for tumor treatment. Further, RGD as a biomimetic peptide can also promote cell adherence to the matrix, prevent cell apoptosis and accelerate new tissue regeneration. Functionalizing material surfaces with RGD can improve cell/biomaterial interactions, which facilitates the generation of tissue-engineered constructs. This paper reviews the main functions and advantages of RGD, describes the applications of RGD in imaging agents, drugs, gene delivery for tumor therapy, and highlights the role of RGD in promoting the development of tissue engineering (bone regeneration, cornea repair, artificial neovascularization) in recent years.

## 1. Introduction

**Figure 1:**
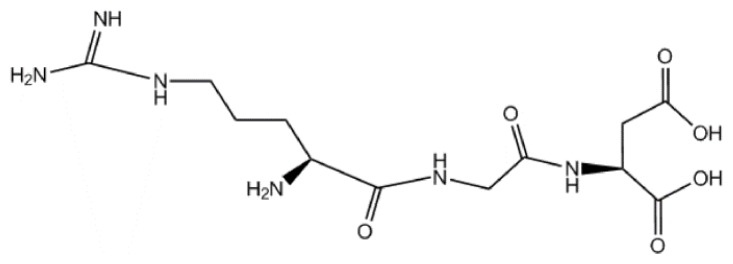
The arginine-glycine-aspartic sequence (RGD).

Arginine-glycine-aspartic (RGD) (Figure 1), is a cell adhesion motif displayed on many extracellular matrix (ECM) and plasma proteins [1]. Since RGD was first identified as specific binding sites for fibronectin (FN) and the FN receptor [2], it has attracted widespread attention and research. Many glycoproteins such as laminin, vitronectin (VN), fibrinogen (Fg), von Willebrand factor (vWF), osteopontin, *etc.* [3], have been found in the ECM, and they are RGD-adhesive proteins. RGD plays an important role in cell recognition and cell adhesion, it has been used into tumor therapy and tissue engineering by recombinant means and some chemical methods.

Hynes [4] had reported that the membrane proteins associated with ECM glycoprotein receptors on the cell surface were called integrins, which were members of the adhesion receptors. The binding of integrins to theirs ligands were dependent on divalent cations to mediate cell-cell and cell-matrix adhesion. Thus, integrins constituted cell adhesion receptors not only for cell-matrix adhesion but also for signaling bidirectionally across the membrane. The large heterodimeric cell surface receptors-integrins were found in many animal species ranging from sponges to mammals [5]. They were involved in fundamental cellular processes such as attachment migration, proliferation, differentiation, and survival. Integrins also contributed to the initiation and progression of many biological diseases such as angiogenesis, thrombosis, inflammation, osteoporosis neoplasia, tumor metastasis and gene expression [6].

RGD-based ligands for integrins are studied in pathology and pharmacology. Furthermore, the RGD-integrin system is exploited to target cell recognition and internalization, which is applied to man-made constructs by mimicking the pathogens. This system enables the study of many aspects (such as diagnostics, therapeutics and the regenerating of transplanted tissue. RGD modified drugs and imaging agents have been investigated and developed by conjugation of the RGD-peptides with a carrier device. The carrier device has been equipped with drug molecules or reporter molecules. RGD-peptides and RGD-mimetics have also been applied to modify liposomes, polymers and peptides by chemical means to improve the biological effects of therapeutic agents. Additionally, RGD-peptides were utilized in gene delivery by viral and non-viral vectors [7]. The surface modification technology with fixed RGD peptides has promoted the application of integrin-mediated cell adhesion to develop tissue engineering, especially for biomaterials.

## 2. The Functions of RGD

### 2.1. The Sequence and the Structure of RGD

The sequence and the structure of RGD-containing peptides have linear and cyclic RGD peptides. However, the cyclic RGD peptides display a higher activity compared to the linear RGD peptides. The advantages of cyclic peptides may be due to a conformationally less flexible structure to resist proteolysis and have the ability to bind with higher affinities to integrin receptors [8,9]. Enwerem *et al.* [10] searched 10 models of cyclic-five-member-ring pentapeptides containing Arginine-Glycine-Aspartic acid (RGD). Among the investigated models, cRGDfV, cRGDfE, cRGDfC, cRGDyV, and cRGDfK represented active RGD peptides. The other five models (cRGDVf, cRGDEf, cRGDCf, cRGDVy, and cRGDKf) were modified (five structures) forms of the previous mentioned RGD species. Among the modified models, cRGDEf and cRGDCf were expected as the new active candidates for the active cyclic RGD pentapeptides.

RGD peptides for scientific research and practical applications have some advantages [11,12]: (i) RGD is much smaller as compared to monoclonal antibodies, and RGD conjugates can have easier access to the tumor tissue; (ii) the use of RGD minimizes the risk of immune reactivity or pathogen transfer; (iii) the synthesis of RGD peptides is relatively simple and inexpensive, which facilitates translation into the clinic; (iv) the applications of RGD are much wider than folic acid. Not only is RGD used in tumor therapy, but it can also be coupled to material surfaces in controlled densities and orientations.

### 2.2. RGD-Mediated Recognition and Adhesion with Cells

RGD, is a cell recognition and attachment site for a number of extracellular matrix proteins as well as blood and cell surface proteins, and has important regulatory functions in many biological activities. RGD is involved in cell attachment, cell spreading, actin-skeleton formation, and focal-adhesion formation with integrins. These four overlapped reactions are important for transmitting signals related to cell behavior and the cell cycle [13].

**Figure 2:**
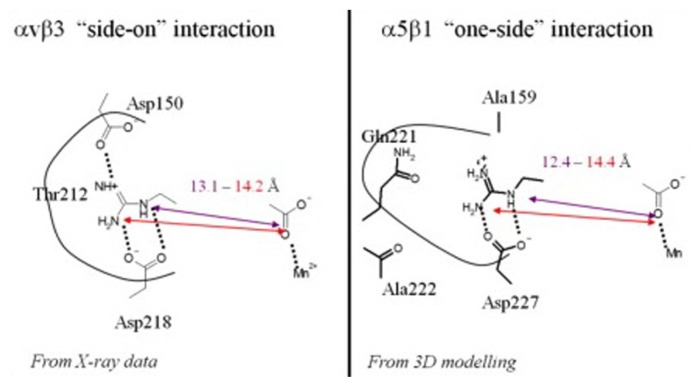
RGD interacting with integrins αvβ3 and α5β3. Reprinted with permission from [15].

Integrins α5β3 and αvβ3 are members of the heterodimeric glycoprotein receptors; they regulate cell-cell and cell-extracellular matrix interactions. Integrin αvβ3 plays a key role in the early stage of angiogenesis; it is expressed at low levels on mature endothelial cells and epithelial cells, but is highly expressed on the activated endothelial cells of tumor neovasculature and other tumor cells, including osteosarcomas, neuroblastomas, glioblastomas, melanomas, lung carcinomas, and breast cancer [14]. The α5β3 and αvβ3 have strong sequence similarity around the RGD binding site, but α5β3 is recognized by RGD through “one-side” interaction while αvβ3 is through “side-on” interaction [15]. The two interactions reveal subtle differences in the location of the conserved Asp in the β propeller groove of the respective α subunits as depicted in Figure 2. That is, in αv, the conserved Asp218 is at the entry of a shallow groove and located on the side while Asp150 is present on the opposite side of this pocket; Asp150 offers an extra H-bond interaction opportunity. Both Asp residues provide a particularly strong interaction by interacting with the arginine. A Thr212 occupies the bottom of the groove. However, in α5, Asp227 occupies the same location at the entry of the pocket as Asp218 (αv), but Asp150 is changed for a small non polar Ala159 and Thr212 in the bottom is replaced by a larger Gln221 in α5. Integrin αIIbβ3 as αll osteric receptor for fibrinogen, only the activated and high-affinity αIIbβ3 interacts with soluble fibrinogen by being dependent on the RGD [16]. In addition, RGD also recognizes α3β1, α8β1, αvβ1, αvβ6, and αvβ5 of the integrin family [17]. The RGD recognition motif makes two key and conserved interactions with both α and β subunits to mediate ligand binding. The guanidine function of arginine is engaged in a bidentate salt bridge with a highly conserved aspartic acid residue in the α subunit of the receptor. The carboxylate group of the aspartic acid coordinates with the metal-ion dependent adhesion site; the adhesion site is located in β subunit of the receptor [18].

In tissue engineering, efficient immobilization of the RGD peptide on the biomaterial surface is important. Cells make contact with cell surface receptors with adhesion in the ECM. It has been reported that the RGD peptide increased cell adhesion and had been used as an artificial ECM protein to induce specific cellular responses and to promote new tissue formation [12].

## 3. The Applications of RGD

Because integrins participate in cell-cell adhesion, cellular differentiation, migration, and attachment to the ECM, RGD peptides are extensively used in many diverse physiological and pathological processes, which mainly focus on the diagnosis and treatment of tumors, the development of anti-cancer drugs and antithrombotic drugs, and tissue engineering (bone regeneration, cornea repair, and artificial neovascularization, *etc.*).

### 3.1. Radiolabelled RGD Peptides for Tumor Imaging and Diagnostics

Tumor imaging has high specificity and sensitivity through conjugation of the RGD-peptide to imaging agents. This important technology has been widely used for the early diagnosis and differential diagnosis of tumors, and in clinical analysis and treatment.

**Figure 3:**
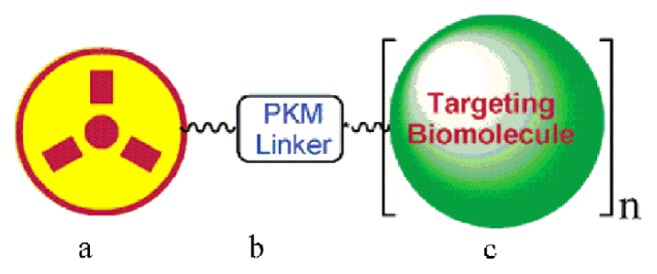
Schematic presentation of the radiopharmaceuticals design. (**a**) Radiometal chelate (e.g., 18F-containing synthon); (**b**) PKM (pharmacokinetic modifying) linker; (**c**) The targeting biomolecule (BM = RGD peptide). Reprinted with permission from [19]. Copyright (2006) American Chemical Society.

**Figure 4:**
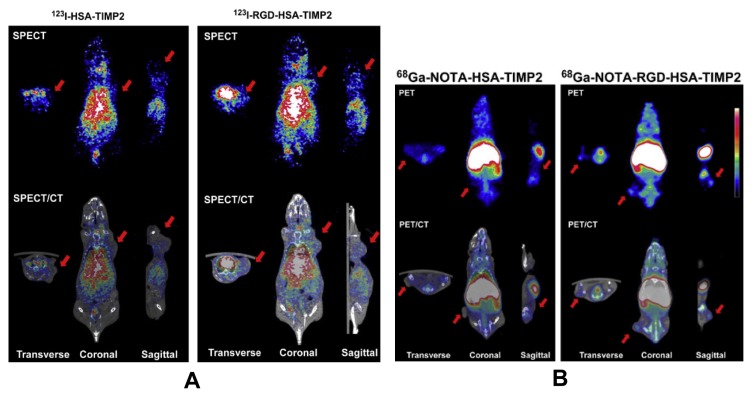
The SPECT and SPECT/CT images of ^123^I-HSA-TIMP2 (**A**, **left**) and ^123^I-RGD-HSA-TIMP2 (**A**, **right**) and the PET and PET/CT images of ^68^Ga-NOTA-HSA-TIMP2 (**B**, **left**) and ^68^Ga-NOTA-RGD-HSA-TIMP2 (**B**, **right**). Red arrows indicated the U87MG tumor. Reprinted with permission from [21].

**Figure 5:**
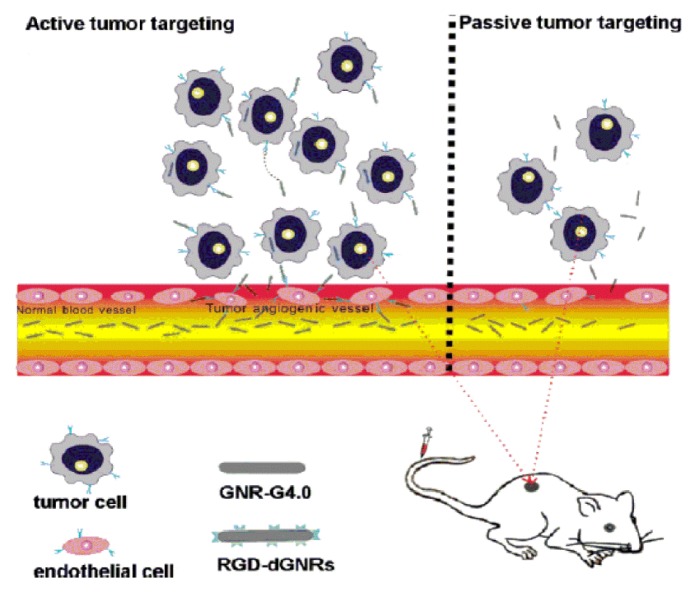
The possible therapeutic mechanism of RGD-dGNR nanoprobes. Reprinted with permission from ([23]). Copyright (2010) American Chemical Society.

RGD has a relatively high and specific affinity for αvβ3 integrins over-expressed in tumour neovasculature. An integrin αvβ3-targeted radiotracer design was simply visualized ([19]; see Figure 3). The RGD peptide (targeting biomolecule) served as a vehicle to carry the radionuclide to the integrin αvβ3 overexpressed on tumor cells. Some progress in tumor-targeted imaging was made by single photon emission computed tomography (SPECT), positron emission tomography (PET), near-infrared fluorescence (NIRF), molecular magnetic resonance imaging (MRI) or photoacoustic imaging with the aid of RGD [20]. Choi *et al.* [21] have synthesized a fusion protein, cyclic arginine-glycine-aspartate (RGD)-HSA-TIMP2. This protein composite was labeled with ^123^I- and ^68^Ga and was evaluated for *in vivo* tumor imaging using SPECT and PET. In SPECT and SPECT/CT images of ^123^I-HSA-TIMP2 (Figure 4(A), left) and ^123^I-RGD-HSA-TIMP2 (Figure 4(A), right) for 4 h after injection, it was observed that ^123^IHSA-TIMP2 was not absorbed by the mice bearing U87MG xenografts, but the level of tumor uptake of ^123^I-HSA-RGD-TIMP2 was slightly higher. In PET and PET/CT images of ^68^Ga-NOTA-HSA-TIMP2 (Figure 4(B), left) and ^68^Ga-NOTA-RGD-HSA-TIMP2 (Figure 4(B), right) for 3 h after injection, the level of tumor uptake of ^68^Ga-NOTA-RGD-HSA-TIMP2 was slightly higher than that of ^68^Ga-NOTA-HSA-TIMP2, when no tumor uptake of ^68^Ga-NOTA-HSA-TIMP2 was observed (Figure 4). These results demonstrated that the new fusion protein had potential not only as an anticancer agent but also as a radioligand for the diagnosis of tumors. Zerda *et al.* [22] showed a contrast agent for photoacoustic imaging of tumors; this contrast agent consisted of single-walled carbon nanotubes conjugated with cyclic RGD peptides. The results showed that these targeted nanotubes to mice bearing tumours displayed eight times greater photoacoustic signal in the tumour than mice injected with non-targeted nanotubes. Li *et al.* [23] reported a class of safe and effective RGD-conjugated dendrimer-modified gold nanorods (RGD-dGNRs). These nanoprobes were verified to have great potential in tumor targeting, imaging, and selective photothermal therapy. The possible therapeutic mechanisms were summarized based on the large number of experiments (Figure 5). RGD-dGNR nanoprobes that were distributed into the whole body of mice with tumors (mainly entered into liver and tumor vessels), could target and bind with the αvβ3 integrins on the surface of melanoma A735 cells and inner walls of tumor vessels. RGD-dGNR damaged vascular endothelial cells and blocked the blood flow in tumor vessels by absorbing NIR laser and transferring laser energy into heat under NIR laser irradiation, which could result in the necrosis or disappearance of tumor tissues via inhibiting the newborn vessels in tumor tissues, and blocking supply chains of nutrients and oxygen to starve tumor cells. Park *et al.* [24] synthesized Gd-DOTA-RGD, as a potential tumor-target for MRI. This complex exhibited higher R1 relaxivity and moderately good specificity for the αvβ3 receptor in hepatocellular carcinoma in Hras12V transgenic mice. Wu *et al.* [25] showed that near infrared (NIR) fluorescent dyes conjugated cyclic RGD peptides could be used for noninvasive NIRF imaging to detect and semi-quantify tumor integrin expression.

Now, the method using radiolabelled RGD peptides for imaging has been translated from the laboratory to the clinic, and the clinical data is critical for the ultimate clinical value of imaging of integrin expression. Thus, large-scale trials using radiolabelled RGD peptides within the context of response assessment or evaluation of patient prognosis are warranted to define the ultimate role of imaging of integrin expression in the clinic.

### 3.2. RGD Inhibiting Tumor

#### 3.2.1. RGD Peptides Affecting Tumor Cell Adhesion and Migration

Angiogenesis-associated integrin αvβ3 represents an attractive target for therapeutic intervention and plays a key role in tumor-induced angiogenesis and tumor growth because of highly up-regulated angiogenic endothelium cells. Kang, *et al.* [26] investigated that RGD-modified salmosin (with salmosin extracted from snake venom), markedly inhibited B16F10 melanoma cell adhesions to the extracellular matrix proteins and hampered B16F10 melanoma cell invasion through a matrigel-coated filter. The inhibition by salmosin was caused by blocking integrins expressed on the surface of B16F10 melanoma cells. This confirmed that RGD-modified salmosin could significantly inhibit solid tumor growth. The immunomodulators, phorbol 12-myristate 13-acetate (PMA) was able to inhibit SNB-19 and T98G cell attachment to fibronectin and vitronectint only by the addition of the tripeptide RGD [27]. Mitjans, *et al.* [28] characterized the therapeutic properties of RGD peptides and monoclonal antibody (MAb) *in vivo* that acted as αv antagonists for blocking human melanoma tumor growth. The Cyclic RGD peptide targeted MAb could successfully inhibit αv integrin–mediated cellular adhesion and induce detachment of previously substrate-attached tumor cells.

#### 3.2.2. RGD Peptides Inducing Tumor Cell Apoptosis

Anuradha *et al.* [29] reported that RGD triggered apoptosis at a concentration of 1 mmol/L by activating caspase-3, leading to DNA fragmentation and cell death; this showed that the RGD peptide could induce tumor apoptosis. Chen *et al.* [30] found that the synthetic RGD-tachyplesin inhibited the proliferation of TSU prostate cancer cells and B16 melanoma cells as well as endothelial cells in a dose-dependent manner *in vitro* and reduced tumor growth *in vivo*. In addition to RGD, the peptide sequence of RGDS, RGDF *etc.* could also induce apoptosis of tumor cells in some experiments [31,32].

#### 3.2.3. RGD Peptides Inhibiting Tumor Angiogenesis

The αv-integrins (αvβ3, αvβ5) regulate the contact of activated endothelial cells to proteins of the extracellular matrix during tumour angiogenesis, which is a prerequisite for survival of endothelial cells. Eliceiri and Cheresh [33] investigated a methylated cyclic RGD-peptide that, as an αv-integrin antagonist, had a disastrous impact on angiogenesis, microcirculation, growth and metastasis formation of a solid tumor *in vivo*; the inhibition of αv-integrins by a cyclic RGD-peptide resulted in significant reduction of functional vessel density, retardation of tumour growth and metastasis *in vivo*. Chavakis *et al.* [34] confirmed that the RGD peptide could completely inhibit the pro-angiogenic role of early vascular endothelial growth factor (VEGF), and even a low dose had the necessary anti-degradation ability to achieve a therapeutic effect.

### 3.3. RGD-Modified Carriers

#### 3.3.1. RGD-Modified Gene Carriers Treating Tumor

**Figure 6:**
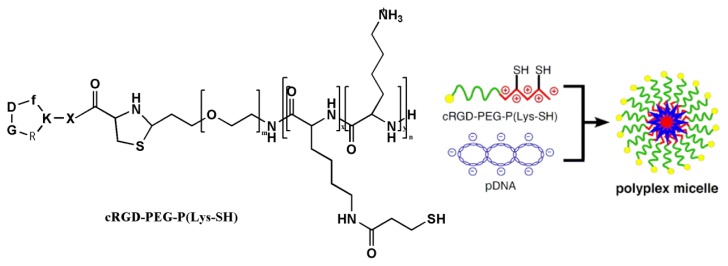
Structure of cRGD-PEG-P(Lys-SH) and its polyplex micelle. Reprinted with permission from [35].

DNA complex-coated RGDs were prepared by covalent conjugation of the RGD-peptide to the various combinations of cationic polymers, lipids and peptides, after which the new RGD-modified compounds were applied to plasmid DNA and other short interfering RNA (siRNA). These carriers have been reported to be used for RGD-modified non-viral gene carriers (Table 1). Vachutinsky *et al.* [35] described the preparation of targetable polyplex micelles through ion complexation of the polymers (c(RGDfK)-PEG-P(Lys-SH)) with plasmid DNA (Figure 6). These RGD-modified cross-linked polyplex micelles carrying sFlt-1 plasmid DNA could inhibit tumor growth. The therapeutic activity of these micelles was achieved by their being accumulated in the tumor and interacting with endothelial cells and enhancing intracellular uptake through receptor-mediated endocytosis.

In addition, viruses, as efficient vectors, have also been used for introducing genetic material into mammalian cells. RGD-modified viruses for gene delivery were reported in Table 2, they could redirect interference genes to tumour cells or angiogenic blood vessels by introducing the additional RGD-peptides into adenoviruses.

#### 3.3.2. RGD-Modified Drugs for Target Therapy

Since the discovery of the RGD motif as a potent ligand of cell surface integrins (mainly αvβ3), RGD-mediated small molecule and RGD-containing therapeutic peptides and proteins, have been used to control drug bio-distribution, and have become employed as agents for cell targeting and endosomal delivery [47]. Because of integrin abundance in endothelial cells, and the vascularization of tumoral tissues, RGD-mediated drug delivery is of special interest in cancer therapies.

RGD-modified PEGylated polyamidoamine (PAMAM) dendrimer with doxorubicin (DOX) conjugated by acid-sensitive cis-aconityl linkage (RGD-PPCD), has been demonstrated to have a significantly prolonged half-life, and exhibited higher accumulation in brain tumor as compared to normal brain tissue. More importantly, this novel RGD-mediated drug demonstrated increased tumor targeting by binding with the integrin receptors overexpressed on tumor cells, and controlled release of free DOX in weakly acidic lysosomes [48]. Wang *et al.* [49] had also synthesized a novel dextran-oleate-cRGDfK conjugated for the self-assembly of this nanodrug; *in vitro* assays proved that this nanodrug could directly and selectively target PTX to MDA-MB-231 cells for effective cell internalization, displaying anti-tumor characteristics superior to Taxol formulation. Therefore, the new DO-cRGDfK conjugate nanodrug could be utilized for effective drug or gene delivery nanoparticles to curb malignant metastatic cancer cells. The more various RGD-modified drugs constructs and descriptions are summarized in Table 3.

### 3.4. RGD Used in Tissue Engineering

Tissue engineering is a new discipline that has been developed in recent years, in which biological substitutes are used to repair, maintain or improve the function of biological tissues through the application of biology and engineering principles. An important concept in tissue engineering is that implanted substrates should not only provide structural support for damaged tissues, but also integrate with these tissues and promote regeneration in the surrounding area. RGD effectively promotes the attachment of numerous cell types to a plethora of diverse materials [12].

**Figure 7:**
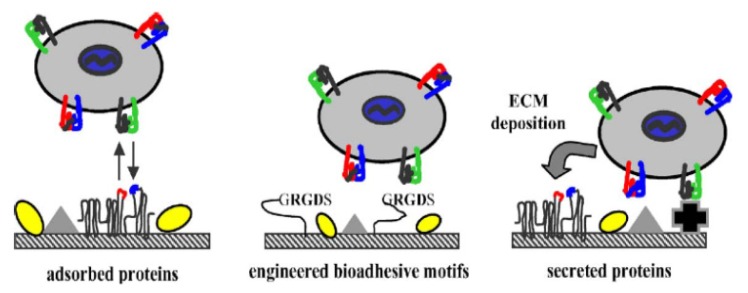
Mechanisms controlling cell adhesion to biomaterials. Reprinted with permission from [58].

Integrin receptors can recognize and interact with the RGD motif, and the functionality of RGD can be maintained after processing and sterilization steps. Modification with fixed RGD peptides on biomaterial surfaces promotes the application of integrin-mediated cell adhesion, and thus encourages the development of tissue engineering. Integrins can control cell adhesion to biomaterial surfaces by interacting with adhesive extracellular ligands, and the mechanisms of this process may be through adsorbing, engineering and depositing as follows: (i) cells adsorb adhesive extracellular ligands (RGD) on biomaterial surfaces with integrins mediating; (ii) biomaterial is engineered at the interface by bioadhesive motifs (RGD) being adsorbed; (iii) more bioadhesive ligands are deposited by cells (Figure 7) [58].

Recently, Michal *et al.* [59] demonstrated that RGD-immobilized alginate scaffolds were beneficial to cardiac tissue engineering, because the immobilized RGD peptide promoted cell adherence to the matrix, prevented cell apoptosis, increased cell survival and recovery, and accelerated cardiac tissue regeneration. Other approaches for RGD-mediated modifying of biomaterials are listed in Table 4.

#### 3.4.1. Bone Regeneration

RGD-based ligands of integrins act as anchor points, because the actin cytoskeleton can bind to these anchor points and form structural components of the cell. The extracellular adhesive proteins, especially the RGD, promote cell adhesion and migration, which play particularly important roles in the osseointegration behavior of osteoblasts. The improved osseointegration observed on RGD-coated surfaces has stimulated recent efforts to attach RGD to oxide coated metals and their alloys such as Ti and Ti-6Al-4V. Chen *et al.* [64] had studied the combined effects of laser micro grooving and RGD-coating on the osseointegration of Ti-6Al-4V pins *in vivo*. The results demonstrated that immobilization of RGD peptides on titanium (Ti) surfaces enhanced implant bone healing by promoting early osteoblastic cell attachment and subsequent differentiation by facilitating integrin binding.

#### 3.4.2. Cornea Repair

Cornea integrity is very critical for our eyes. Corneas may be damaged when cornea endothelial cells become less abundant with age or disease [67]. Developments in tissue engineering have the potential to repair corneas. RGD-mediated surface modification facilitated cell attachment, proliferation, alignment and expression of both collagens (type I and V) and proteoglycans (decorin and biglycan). Gil *et al.* [68] had demonstrated that the RGD-coupled silk human cornea structure could be bio-functionalized by integrating corneal stroma tissue with proteoglycan-rich extracellular matrix. This biomimetic approach to replicate the structural hierarchy and architecture of corneal stromal tissue may become a useful strategy for engineering human cornea. Further, this approach can be exploited for other tissue systems to be repaired.

#### 3.4.3. Artificial Neovascularization

The demand for small-diameter vascular grafts for cardiovascular disease is growing, so it is necessary to develop substitutes with bio-functionalities. RGD-modified grafts exhibited an improved inhibition of platelet adhesion, which enhanced cell infiltration, endothelium formation, smooth muscle regeneration and patency, the as-prepared PCL-RGD graft may be a promising candidate for the small-diameter vascular grafts [60].

## 4. Conclusions

In summary, the RGD-peptide sequence, as the specific recognition site of interaction between integrins and their ligands, has strong potential in applications for cancer therapy and tissue engineering. However, the functional mechanisms of RGD in cancer treatment and tissue engineering should be further investigated in order to close the gap between the experimental data and their translation to clinical application.

## Figures and Tables

**Table 1:** RGD-modified non-viral carries for gene delivery [7].

Carriers	Production	Gene	Experimental model
Polymer	RGD-PEG-PCL	siRNA	B16F10-luc2 lung metastatic [36]
	RGD-PEG	PEG-PEI/DNA complexes	NIH3T3 cells [37]
	RGD-PEG-PEI	Plasmid DNA	Intracranial glioblastoma [38]
	RGD-PEG-Suc	Plasmid DNA coding luciferase	Melanoma cell [39]

*Lipid*	Polymerized lipid nanoparticle	ATP-Raf	M21-L/CT26 colon carcinoma [40]

*Liposomes*	RGD-SSL-DOX liposomes	RGD-Lipo-siRNA(MDR1)	Breast cancer MCF7/A cells [41]
	RGD-PEGylated liposomes	siRNA	Pigment epithelial cells [42]

*Peptides*	RGDGWK-lipopeptde	Anti-cancer p53 gene	B16F10 tumor [43]
	RGD-HK-branched peptides	siLacZ, siLuciferase	MDA-MB-435c, MCF7 [44]

**Table 2:** RGD-modified viruses for gene delivery.

Production	Gene	Experimental model
RGD4C/AAV/phage hybrid	GFP or Luc reporter genes	Human M21 Melanoma Human U87 glioblastoma [45]
Ad/CD-PEG500-RGD	shRNA	Various cancer cells [3]
(Adenovirus) Ad-RGD	Firefly luciferase gene	Mouse TS cells [46]

**Table 3:** RGD modified drugs.

Category	Drug	Production	Experimental model
*small molecule drugs*	Paclitaxel	PTX-RGD/Tf-NPs (nanoparticles)	HeLa cells [50]
	Doxorubicin	RGD-PEG-PAMAM-DOX	C6 glioma cells [51]
	Combretastatin A-4 (CA-4) and doxorubicin (Dox)	RGD-CA-4 and Dox liposomes	B16 and B16F10 melanoma cells [52]
	Docetaxel	RGD-PEG-LP-DC (Liposomes)	BT-20 and MDA-MB-231 cells [53]
*therapeutic proteins and peptides*	Fibulin-5	RGD-fibulin-5	A549, H1299 and H460 cells [54]
	tTf	(RGD)3/tTF	H460 lung cancer cells [55]
	The angiogenic factor Del1	RGD-Mediated angiogenic factor	Human umbilical vein endothelial cells [56]
	Osteopontin	RGD-containing Osteopontin	Avian osteoclast-like cells [57]

**Table 4:** RGD-peptide modified biomaterials.

Category	Biomaterials	Compound	Experimental model
*Polymer*	Alginate scaffolds	RGD-immobilized alginate scaffolds	Cardiac cell [59]
	PCL (polycaprolactone)	RGD-PCL	Vascular grafts rabbit carotid artery [60]
	PCL	RGD-modified 3D-PCL	Bone marrow stromal cells [61]
	Poly (ethylene imine)-poly(2-vinyl-4,4-dimethylazlactone)	RGD-PEI/PVDMA	Human corneal epithelial cell [62]
*Inorganic materials*	Hydroxyapatite (HA)	RGD-coated HA	Rat tibiae [63]
	Ti6-Al-4V pins	RGD-coated Ti6-Al-4V pins	Rabbit femurs [64]
*Proteins*	Spider silk	RGD-modified spider silk	BALB/3T3 mouse fibroblasts [65]
	Nephronectin	RGD nephronectin	Cardiomyocytes [66]
